# Preventive Effect of Alkaloids from *Lotus plumule* on Acute Liver Injury in Mice

**DOI:** 10.3390/foods8010036

**Published:** 2019-01-19

**Authors:** Bihui Liu, Ji Li, Ruokun Yi, Jianfei Mu, Xianrong Zhou, Xin Zhao

**Affiliations:** 1 Chongqing Collaborative Innovation Center for Functional Food, Chongqing University of Education, Chongqing 400067, China; liubh@foods.ac.cn (B.L.); yirk@cque.edu.cn (R.Y.); mujianfei@foods.ac.cn (J.M.); zhouxr@foods.ac.cn (X.Z.); 2 Chongqing Engineering Research Center of Functional Food, Chongqing University of Education, Chongqing 400067, China; 3 Chongqing Engineering Laboratory for Research and Development of Functional Food, Chongqing University of Education, Chongqing 400067, China; 4 College of Biological and Chemical Engineering, Chongqing University of Education, Chongqing 400067, China; liji@foods.ac.cn

**Keywords:** *Lotus plumule*, antioxidant, liver injury, gene, mice

## Abstract

*Lotus plumule* is a traditional Chinese food that can be used as a beverage. In this study, three kinds of *Lotus plumules* from different regions of China were selected to observe the preventive effects of extracted alkaloids on CCl_4_-induced liver injuries. Animal experiments revealed that alkaloids extracted from *Lotus plumules* decreased the serum AST (aspartate aminotransferase), ALT (alanine aminotransferase), and TBIL (total bilirubin) levels, enhanced SOD (superoxide dismutase) activity, and reduced MDA (malondialdehyde) level in the liver tissues of mice with liver injury. H&E observation confirmed that alkaloids from *Lotus plumules* could alleviate CCl_4_-induced injuries of liver tissues and inhibit the inflammatory effect on hepatocytes. Further qPCR experiments also demonstrated that alkaloids from *Lotus plumules* upregulated the expression of IκB-α (inhibitor of NF-κB alpha), Cu/Zn-SOD (copper/zinc superoxide dismutase), Mn-SOD (manganese superoxide dismutase), and CAT (catalase) mRNA and downregulated TNF-α (tumor necrosis factor alpha) and NF-κB (nuclear factor kappa B) expression in the liver tissues of mice with liver injury. All three kinds of alkaloids from *Lotus plumules* could prevent CCl_4_-induced liver injuries by regulating the levels of oxidative stress and inflammation in mice, and the therapeutic effect was comparable to that of silymarin, the medicine commonly used in the treatment of liver diseases. In summary, alkaloids from *Lotus plumules* contain bioactive substances with hepatic protective efficacy and possess potential application value in the field of functional food.

## 1. Introduction

*Lotus plumule* is the green radicle in the middle of mature seeds of Nymphaeaceae plant lotus. In addition to being a traditional Chinese medicine, it is more commonly used as a beverage in China [1]. *Lotus plumule* is rich in alkaloids and sterols, and it also contains water-soluble polysaccharides and a variety of micronutrients [2]. In traditional Chinese medicine, *Lotus plumule* has the effects, such as nourishing Yin, suppressing hyperactive yang, purging fire, eliminating toxins, clearing heat, cooling the blood, promoting the secretion of saliva and body fluid, regulating Qi, protecting the stomach and the liver, improving vision, and inducing ataraxia [3]. Modern medical science also proves that *Lotus plumule* can lower blood pressure by regulating blood vessels and smooth muscles [4]. Studies have also shown that *Lotus plumule* has a good effect of clearing oxygen free radicals. It can capture lipid peroxide free radicals generated in the process of oxidation, stimulate the enzymatic antioxidant mechanisms, activate and enhance the activities of antioxidant enzymes, such as SOD (superoxide dismutase) and CAT (catalase), and block the oxidative chain reaction of free radicals, thereby rapidly clearing free radicals in living organisms [5].

The liver is the largest solid organ in the abdominal cavity and plays an important physiological role in the human body. Hepatic arteries and portal veins provide abundant blood supply, since hepatocytes are vulnerable to hypoxia, and bile ducts accompanying blood vessels transport bile. However, the liver is easily damaged due to its large size and fragility and causes intra-abdominal hemorrhage or bile leakage, resulting in hemorrhagic shock or biliary peritonitis that has serious consequences [6]. The oxidative stress induced by reactive oxygen species is the common pathophysiological basis of many hepatic diseases. Oxidative stress mainly changes the function of biofilm, covalently binds to biomacromolecules, and hampers the activity of enzymes by initiating membrane lipid peroxidation, which in combination with cytokines, such as TNF-α and NF-κB, results in various degrees of liver injury. Oxidative stress plays an important role in the progress of fatty liver, viral hepatitis, and liver fibrosis [7,8]. CCl_4_ is a common inducer of liver injuries. A large number of harmful free radicals will be generated by the activation of liver microsomal cytochrome P-450 after CCl_4_ enters the body, which increases calcium ion influx by reducing the activity of calcium pump in the endoplasmic reticulum, promotes the production of inflammatory cytokines in hepatocytes, aggravates inflammation, induces the activation of phosphorylase on the membrane of hepatocytes, causes membrane peroxidation, destroys hepatocyte membrane, and leads to liver injuries [9].

Alkaloids are a class of alkaline organic compounds containing nitrogen in the plants. Plant alkaloids, such as *Coptidis* alkaloids, alkaloids in *Caulis mahoniae*, and *Clivia miniata* alkaloids all show strong antioxidant effects. These plant alkaloids have bacteriostatic activities and protective effects on liver and kidney functions [10,11,12]. Studies have also shown that alkaloids from *Lotus plumule* have various health care functions, such as anti-hypertension, anti-oxidation and protection of the myocardium [13].

In this study, to determine the effect of *Lotus plumule* alkaloids, three *Lotus plumules* from China’s main *Lotus plumule* producing areas were selected, the vast majority of *Lotus plumule* varieties in China come from the three *Lotus plumules* selected in this study, so we chose these three representative lotus seed hearts for the observation of preventive effect of *Lotus plumule* alkaloids on acute liver injuries in mice. An acute liver injury model was established with carbon tetrachloride. The preventive effect on liver injury was verified by examining biochemical indices and mRNA expressions. Results of this study will provide the theoretical basis for the further precision application of alkaloids from *Lotus plumule*, which will contribute to the development and application of these agents.

## 2. Materials and Methods

### 2.1. Extraction of Alkaloids from Lotus Plumule

Ultrasound-assisted extraction with higher extracting activity was used. The *Lotus plumules* were pulverized after drying at 90 °C for 2 h. 100 g of *Lotus plumule* powder was accurately weighed and placed in a plugged conical flask. 500 mL 65% ethanol was added, and the sealed conical flask was fixed in an ultrasonic cleaner. Extraction was carried out by 220 W ultrasound at 30 °C for 45 min, and then extracted liquid was filtered. The residue was mixed with 500 mL 65% ethanol and extracted by ultrasound once more under the same conditions. Extracted liquids were collected and filtered by a Brinell funnel. The filtrate was concentrated and dissolved in the appropriate amount of water. A hydrochloric acid solution was used to adjust the pH level to 1–2. After filtration, extracted liquids were adjusted to pH 9–10 with ammonia solution. After standing, extracted liquids were centrifuged at 3000 r/min for 10 min and alkaloids from *Lotus plumule* were obtained after drying at 50 °C [14].

### 2.2. Determination of Alkaloid Content

Standard neferine was used as a reference substance for determining the content of alkaloids extracted from *Lotus plumules*. The standard neferine was weighed and dissolved in 0.1 mol/L hydrochloric acid solution to prepare neferine solution at the mass concentrations of 15, 20, 25, 30, 35, 40, 45 and 50 μg/mL. The absorbance (A) of the above solutions was measured at the wavelength of 276 nm. The standard curve was obtained with A serving as the ordinate versus mass concentration of neferine (ρ) as the abscissa [15].

### 2.3. Animal Experiments with Mice

Sixty 6-week-old male SPF KM mice (Chongqing Medical University, Chongqing, China) were kept for adaptation to the environment for 1 w and were divided into six groups: Normal group, control group, silymarin group and groups of total alkaloids of lotus seeds from Hunan Province (LSA-HN), Jiangxi Province (LSA-JX), and Hubei Province (LSA-JX), with 10 mice in each group. Mice in the normal and control groups were gavaged with normal saline. Mice in the silymarin group were gavaged with 200 mg/kg silymarin (Sigma-Aldrich, Inc., St. Louis, MO, USA), while mice in the LSA-HN, LSA-JX and LSA-HB groups were gavaged with LSA-HN, LSA-JX and LSA-HB at the concentration of 400 mg/kg, respectively. The above administrations lasted 10 d. At Day 10, mice in the control, silymarin, and alkaloid groups were intraperitoneally injected with vegetable oil--CCl_4_ solution (0.8% CCl_4_ solution, injection dose of 0.1 mL/10 g) 1 h after gavage, whereas mice in the normal group were intraperitoneally injected with the same dose of vegetable oil alone [16]. After intraperitoneal injection of CCl_4_ solution, all experimental mice were fasted for 24 h and the liver tissue and blood of mice were collected for further experiments after sacrifice. The liver tissue index of mice was determined as organ mass (g)/body mass (kg) × 100. This study was conducted in accordance with the Declaration of Helsinki, and the protocol was approved by the Ethics Committee of Chongqing Collaborative Innovation Center for Functional Food (201802001B).

### 2.4. Detection of Serum AST, ALT and TBIL Levels

The obtained plasma was centrifuged at 4000 rpm for 10 min, and the upper serum was collected. Then the levels of aspartate transaminase (AST), alanine transaminase (ALT), and total bilirubin (TBIL) in the serum of mice were measured by the kits (Nanjing Jiancheng Bioengineering Institute, Nanjing, China).

### 2.5. Detection of SOD and MDA Levels in Liver Tissues

The 10% homogenate was obtained from the liver tissue of the mice and centrifuged at 4000 rpm for 10 min. The supernatant was collected and the levels of superoxide dismutase (SOD) and malondialdehyde (MDA) in the liver tissue were measured by the kits (Nanjing Jiancheng Bioengineering Institute, Nanjing, China).

### 2.6. Pathological Observation of Liver Tissue

About 0.5 cm^2^ of mice liver tissue was fixed in 10% formalin solution for 48 h. The liver tissue was then dehydrated, cleared, waxed, embedded, sliced, and stained with H & E stain. The morphological changes in the liver tissues were observed under an optical microscope (BX43, Olympus, Tokyo, Japan).

### 2.7. Quantitative PCR (qPCR) Assay

The tongue tissues of the mice were pulverized. Total RNA (Thermo Fisher Scientific, Inc., Waltham, MA, USA) in the tongue tissues was extracted with RNAzol and the concentration of extracted total RNA was diluted to 1 μg/μL. Then 5 μL of diluted total RNA solution was collected and used for reverse transcription to obtain the cDNA template according to the kit instruction. 2 μL cDNA template was mixed with 10 μL SYBR Green PCR Master Mix and 1 μL upstream and downstream primers (Table 1). The reaction was performed at 95 °C for 60 s, then 40 cycles at 95 °C for 15 s, 55 °C for 30 s, and 72 °C for 35 s. Finally, the detection was carried out under the conditions at 95 °C for 30 s and 55 °C for 35 s. The relative expressions of genes were calculated by 2^−ΔΔCt^ method with GAPDH serving as the internal reference [17].

### 2.8. Statistical Analysis

Three parallel experiments were performed on the serum and tissue indices of each mouse, and the average values were obtained. Statistical software SAS9.1 (SAS Institute Inc., DriveCary, NC, USA) was used to analyze the data. One-way ANOVA according to Duncan’s multiple-range test was used to judge whether there were significant differences among groups at the level of *p* < 0.05. If the superscript letters of data using a to d were the same, it meant that there was no significant difference between the data marked by the same letter at *p* > 0.05 level; if the superscript letters of data using a to d were different, it means that there was a significant difference between the data marked by different letters at *p* < 0.05 level.

## 3. Results

### 3.1. Content of Alkaloids Extracted from Lotus Plumules 

Neferine served as the standard substance and the regression equation was A = 59.849ρ + 0.8694 (*R*^2^ = 0.9988). According to the regression curve, the purity of alkaloid extracts in the LSA-HN, LSA-JX, and LSA-HB groups were similar (Table 2). It can be seen that the substances that played core biological roles in the further animal experiments were the components of alkaloids from *Lotus plumules*.

### 3.2. Body Weight, Liver Weight and Liver Index of Mice

As shown in Table 3, except that the mice in the normal group had slightly higher body weight, body weights of mice in each group were similar, and there was no significant difference in the body weights in each group (*p* > 0.05). The liver weights of normal mice were lowest, while the liver weights of control mice were significantly higher than those in other groups (*p* < 0.05). The liver weights of mice with CCl_4_-induced liver injuries were decreased with the gavage of alkaloids from *Lotus plumules*. The results of the liver index are similar to those of liver weight. The control group had the highest liver index. The liver index of mice with CCl_4_-induced liver injuries was significantly lower than that of the control group (*p* < 0.05) after gavage with alkaloids from *Lotus plumules*.

### 3.3. Serum Levels of AST, ALT and TBIL in Mice

Mice in the normal group had the lowest serum levels of AST, ALT and TBIL (Table 4). The control group with CCl_4_-induced liver injury showed the opposite trend with the highest levels of AST, ALT, and TBIL. Compared with the control group, silymarin and alkaloid could significantly downregulate the levels of AST, ALT, and TBIL in mice with liver injuries (*p* < 0.05). There was no significant difference in serum indicators among the groups of *Lotus plumule* alkaloids from different regions (*p* > 0.05). The levels of AST, ALT, and TBIL in the silymarin group were lower than those in alkaloid groups from different regions, and were close to those in the normal group.

### 3.4. SOD and MDA Levels in Liver Tissues of Mice 

Mice in the normal group had the highest SOD activity level and lowest MDA activity level in the liver tissue (Table 5). The control group with CCl_4_-induced liver injury showed the opposite trend with the lowest SOD activity level and the highest MDA activity level. Compared with the control group, gavages of silymarin and *Lotus plumule* alkaloids from different regions significantly promoted the SOD activity and reduced the MDA activity in the injured liver tissues of mice (*p* < 0.05). The levels of SOD and MDA in the silymarin group were close to those in the normal group, and there was no significant difference among the groups of alkaloids from different regions (*p* > 0.05).

### 3.5. Pathological Observation of Mice Liver

**Figure 1:**
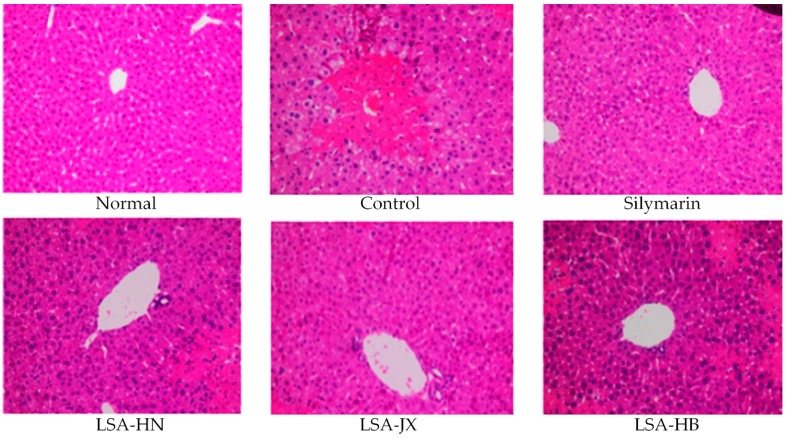
H & E pathological observation of liver in mice. Magnification 100×. Silymarin, mice treated with 200 mg/kg silymarin; LSA-HN, mice treated with 400 mg/kg *Lotus plumule* alkaloids (Hunan, China); LSA-JX, mice treated with 400 mg/kg *Lotus plumule* alkaloids (Jiangxi, China); LSA-HB, mice treated with 400 mg/kg *Lotus plumule* alkaloids (Hubei, China).

The histopathological sections of mouse liver tissues were observed under a microscope (Figure 1). The hepatic lobular structure of mice in the normal group was clear. The hepatocytes arranged radially around the end of the central vein. The proportion of the sinus was normal. The boundary of the hepatic cells was clear, and the nuclei were located in the center of cells. No hyperplasia of fibrous tissues could be found in the portal area. In the control group, the structure of hepatic tissues was disordered, and a wide range of steatosis, vacuolar degeneration, and diffuse necrosis of hepatocytes appeared. Compared with the control group, silymarin and alkaloids from *Lotus plumules* could alleviate the CCl_4_-induced injuries of liver tissues, reducing changes in the hepatic tissue structure and hepatocyte morphology in mice.

### 3.6. Expression of Inflammation-related Genes in Mouse Liver Tissues 

**Figure 2:**
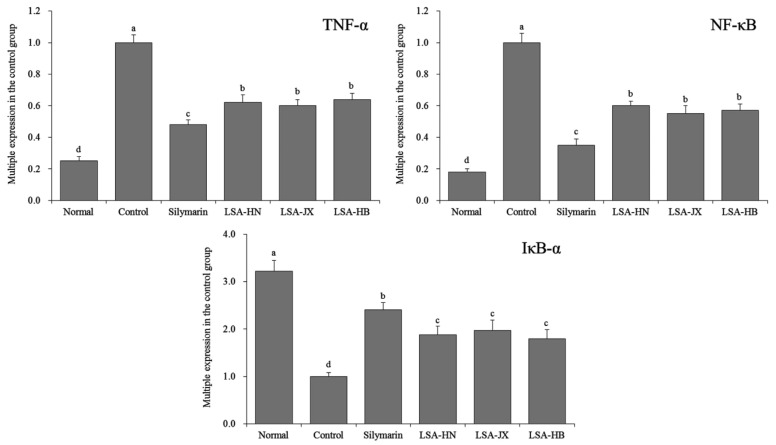
The TNF-α, NF-κB and IκB-α mRNA expression in liver of mice. ^a–d^ Mean values with different superscript letters are significantly different (*p* < 0.05) by the Duncan’s multiple-range test. Silymarin, mice treated with 200 mg/kg silymarin; LSA-HN, mice treated with 400 mg/kg *Lotus plumule* alkaloids (Hunan, China); LSA-JX, mice treated with 400 mg/kg *Lotus plumule* alkaloids (Jiangxi, China); LSA-HB, mice treated with 400 mg/kg *Lotus plumule* alkaloids (Hubei, China).

Figure 2 reveals that the hepatic mRNA expression of TNF-α and NF-κB was weakest, while the expression of IκB-α was strongest in the normal group. The expression of TNF-α and NF-κB increased significantly (*p* < 0.05), whereas the expression of IκB-α decreased significantly (*p* < 0.05) after induction of liver injury by CCl_4_. Silymarin and alkaloids from *Lotus plumules* could significantly (*p* < 0.05) inhibit the upregulated expression of TNF-α and NF-κB, and downregulated expression of IκB-α in the injured liver tissues of mice.

### 3.7. Expression of Oxidation-Related mRNA in Liver Tissues of Mice

**Figure 3:**
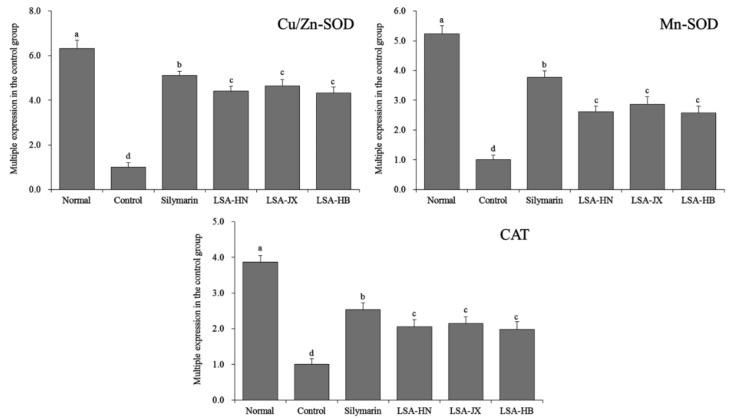
The Cu/Zn-SOD, Mn-SOD and CAT mRNA expression in liver of mice. ^a–d^ Mean values with different superscript letters are significantly different (*p* < 0.05) by the Duncan’s multiple-range test. Silymarin, mice treated with 200 mg/kg silymarin; LSA-HN, mice treated with 400 mg/kg *Lotus plumule* alkaloids (Hunan, China); LSA-JX, mice treated with 400 mg/kg *Lotus plumule* alkaloids (Jiangxi, China); LSA-HB, mice treated with 400 mg/kg *Lotus plumule* alkaloids (Hubei, China).

Figure 3 shows that the hepatic mRNA expression of Cu/Zn-SOD, Mn-SOD, and CAT was strongest in the normal group, while that in the control group was weakest. Silymarin and alkaloids from *Lotus plumules* could significantly upregulate the mRNA expression of Cu/Zn-SOD, Mn-SOD, and CAT in the injured liver tissues of mice.

## 4. Discussion

As one of the five internal organs, the liver plays a very important role. Hepatic injury may threaten the health of the body and is even lethal. At present, the degree of liver injury is clinically assessed with various indices, including the liver index, serum biochemical index, and liver tissue oxidation index. The liver index, also known as liver coefficient, is one of the pathological indicators of liver injury, which has been widely used to evaluate the degree of carbon tetrachloride-induced hepatic injury. The changes in liver weight can directly reflect the damage of liver [18]. The liver is a metabolic organ of mice. The decreased liver weight directly affects the metabolic capacity of animals. The liver is also one of the immune organs in animals [19]. Therefore, measurement of the liver index can directly reflect the structural changes and hepatic functions, and can be used to evaluate the degree of liver injury in mice [20]. Results of this study confirmed that CCl_4_ increased the organ index, and alkaloids from *Lotus plumules* could effectively alleviate increased liver index to a level very close to normal mice, which was comparable to silymarin, the common treatment drug for liver injury.

Hepatocytic membrane permeability increases after CCl_4_ induces liver injury, leading to inflammation of the liver. Transaminases are released from hepatic cells into the blood, and serum levels of ALT and AST increase [21]. The TBIL index reflects the process of the metabolic process of bile components, in which hepatocytes intake indirect bilirubin, combine it with Y and Z proteins, and transport it to the endoplasmic reticulum where indirect bilirubin binds to glucuronic acid to form bilirubin. Therefore, hepatocytes have the function of intaking, binding, and excreting bilirubin [22]. When CCl_4_ injures liver tissue, bilirubin flows back into the blood, leading to an increase in total bilirubin (TBIL) in the serum. Therefore, the change in serum TBIL content can be used to assess the degree of liver injury [23]. Increased levels of AST, ALT, and TBIL indicate exacerbated liver injury. The experimental data of this study also confirmed that CCl_4_ led to increased AST, ALT, and TBIL levels in mice. Alkaloids from *Lotus plumules* could significantly reduce the serum levels of AST, ALT, and TBIL in mice, and the effect was close to silymarin, a drug for liver diseases.

CCl_4_ causes oxidative stress and induces a large number of free radicals in the liver tissues of mice [24]. The activity of SOD can be used to evaluate the degree of liver injury. Regulation and promotion of SOD is the main mechanism of enzymatic antioxidant activity. Therefore, the level of SOD activity reflects the degree of hepatic cell injury [24]. MDA is the metabolic end product of oxidative injury and is accumulated in the body after liver injury. The level of MDA reflects the degree of oxidative damage. It is also a sensitive indicator of liver injury [25]. In this study, alkaloids from *Lotus plumules* could enhance SOD activity and reduce MDA level in the injured liver tissue of mice, thereby preventing CCl_4_-induced liver injury.

TNF-α is a polypeptide medium with extensive biological activities, which mediates liver injury caused by various factors. Liver injury is directly related to the increase of TNF-α [26]. NF-κB is a converging point of various signaling pathways, and plays a key role in various diseases, such as trauma and infection by regulating inflammatory response, and cellular apoptotic genes. There is a NF-κB binding site on the TNF-α promoter. Activated by traumatic stress, endotoxin, and other factors, NF-κB promotes the expression of apoptotic genes and TNF-α. The increased TNF-α activates NF-κB again while mediating apoptosis, which promotes cellular apoptosis and cytokines, such as TNF-α, resulting in a cascade of inflammatory responses centered on NF-κB [27]. 

IκB-α is an inhibitory protein of nuclear factor NF-κB. It binds to NF-κB via an ankyrin repeat motif and covers the nuclear localization sequence (NLS) of its Rel homology domain, which inhibits the activity of NF-κB [28]. In the resting state, two subunits of NF-κB p65, p50 and IκB-α, exist in the cytoplasm in an inactivated state. When the upstream signal activates IKK (IkB kinase), the activated IKK can ubiquitinate, phosphorylate, and degrade IκB-α, which activates the two subunits of NF-κB from the inactivated state and transfers them from the cytoplasm into the nucleus. The two subunits bind to the corresponding inflammation-related genes, initiate the transcription of inflammatory cytokines, and induce inflammation [29]. Therefore, alkaloids from *Lotus plumules* can prevent liver injury by inhibiting the expression of TNF-α, NF-κB, and promoting the expression of IκB-α in liver tissues.

SOD can be divided into three types according to the different metal prosthetic groups it contains. The first one is Cu/Zn-SOD containing metal prosthetic groups of Cu and Zn, which is the most common enzyme and mainly exists in the cytoplasm. The second one is Mn-SOD containing the metal prosthetic group Mn, which exists in the mitochondria of eukaryotic cells and prokaryotic cells. Both Cu/Zn-SOD and Mn-SOD are important antioxidant enzymes that scavenge free radicals in living organisms. They can resist and block the damage caused by oxygen free radicals, promptly repair damaged cells, and reverse cellular injury caused by CCl_4_-induced free radicals [30]. CAT, mainly distributed in the peroxide of cells, can degrade H_2_O_2_ into water and oxygen and clear H_2_O_2_ through a series of metabolic reactions to alleviate tissue inflammation and injury caused by oxidative stress [31]. Alkaloids from *Lotus plumules* show promising efficacy in alleviating liver injury by increasing the levels of antioxidant enzymes (Cu/Zn-SOD, Mn-SOD, and CAT), which is comparable to the efficacy of the drug silymarin.

There are many studies to prove that *Lotus plumules* alkaloids contain only three components, namely, liensinine, isoliensinine and neferine [32,33,34]. Liensinine can inhibit lipid peroxidation in experimental animals [35], and study has confirmed that it has an antioxidant effect [36], thus exerting its influence on inflammation. Isoliensinine also has a good antioxidant effect in vitro [37], and has a significant inhibitory effect on lipid peroxidation in liver tissue [38]. Neferine has a good recovery effect on tissue damage caused by oxygen free radicals, and can also condition the body’s low density lipoprotein and very low density lipoprotein [39]. Liensinine, isoliensinine and neferine have good antioxidant and lipid peroxidation inhibition abilities, which can resist irreversible oxidative stress damage of liver tissue caused by carbon tetrachloride, thus playing a protective role in liver tissue.

## 5. Conclusions

This study showed that the alkaloids from *Lotus plumules* collected from different regions could prevent CCl_4_-induced liver injury by regulating the levels of oxidative stress and inflammation in mice. The experimental results confirmed that alkaloids of *Lotus plumules* from different regions could downregulate serum AST, ALT, and TBIL, enhance SOD activity, and reduce MDA levels in the injured liver tissues of mice. Pathological observations also confirmed that alkaloids from *Lotus plumules* could control the injuries of hepatocytes induced by CCl_4_. Further qPCR experiments also demonstrated that alkaloids from *Lotus plumules* could upregulate the expression of IκB-α, Cu/Zn-SOD, Mn-SOD, and CAT mRNA and downregulate the expression of TNF-α and NF-κB in injured liver tissues of mice. By regulating the above factors, these alkaloids could alleviate liver damage caused by CCl_4_, and the effect was close to the hepatic treatment drug silymarin. Therefore, it can be proved that alkaloids of *Lotus plumules* from food are bioactive substances with good hepatic protective function, as well as promising application value. This study has provided a theoretical basis for further human experiments, and the specific mechanism of its role needs further research.

## Figures and Tables

**Table 1:** Sequences of primers used in this study.

Gene Name	Sequence
TNF-α	Forward: 5′-CGAGTGACAAGCCCGTAGCC-3′
	Reverse: 5′-GGATGAACACGCCAGTCGCC-3′
NF-κB	Forward: 5′-ATGGCAGACGATGATCCCTAC-3′
	Reverse: 5′-CGGAATCGAAATCCCCTCTGTT-3′
IκB-α	Forward: 5′-ATGGCAGACGATGATCCCTAC-3′
	Reverse: 5′-CGGAATCGAAATCCCCTCTGTT-3′
Cu/Zn-SOD	Forward: 5′-AACCAGTTGTGTTGTCAGGAC-3′
	Reverse: 5′-CCACCATGTTTCTTAGAGTGAGG-3′
Mn-SOD	Forward: 5′-CAGACCTGCCTTACGACTATGG-3′
	Reverse: 5′-CTCGGTGGCGTTGAGATTGTT-3′
CAT	Forward: 5′-GGAGGCGGGAACCCAATAG-3′
	Reverse: 5′-GTGTGCCATCTCGTCAGTGAA-3′
GAPDH	Forward: 5′-AGGTCGGTGTGAACGGATTTG-3′
	Reverse: 5′-GGGGTCGTTGATGGCAACA-3′

TNF-α, tumor necrosis factor α; NF-κB, nuclear factor κ -light-chain-enhancer of activated B cells; IκB-α, nuclear factor of κ-light polypeptide gene enhancer in B-cells inhibitor-α; Cu/Zn-SOD, cuprozinc-superoxide dismutase; Mn-SOD, manganese superoxide dismutase; CAT, catalase; GAPDH, glyceraldehyde-3-phosphate dehydrogenase.

**Table 2:** Content of alkaloids extracted from *Lotus plumules*.

LSA-HN	LSA-JX	LSA-HB
65.3% ± 1.23 ^a^	66.7 ± 2.37 ^a^	66.2 ± 2.08 ^a^

^a^ Mean values the date are not efficiently different (*p* > 0.05) by Duncan’s multiple range test. SA-HN, alkaloids of *Lotus plumule* from Hunan, China; LSA-JX, alkaloids of *Lotus plumule* from Jiangxi, China; LSA-HB, alkaloids of *Lotus plumule* from Hubei, China.

**Table 3:** Effects of alkaloids from *Lotus plumules* on body weight, liver weight and liver index of mice with hepatic injury induced by carbon tetrachloride (*N* = 10).

Group	Body Weight (g)	Liver Weight (g)	Liver Index
Normal	32.00 ± 0.92 ^a^	1.44 ± 0.79 ^c^	4.5 ± 0.2 ^c^
Control	29.28 ± 1.57 ^a^	1.89 ± 0.96 ^a^	6.5 ± 0.5 ^a^
Silymarin	29.45 ± 1.53 ^a^	1.70 ± 0.68 ^b^	5.8 ± 0.4 ^b^
LSA-HN	29.29 ± 1.81 ^a^	1.71 ± 0.20 ^b^	5.8 ± 0.5 ^b^
LSA-JX	30.01 ± 1.30 ^a^	1.82 ± 0.15 ^b^	6.1 ± 0.4 ^b^
LSA-HB	29.9 ± 71.29 ^a^	1.80 ± 0.13 ^b^	6.0 ± 0.4 ^b^

Values presented are the mean ± standard deviation (*N* = 10/group). ^a–^^c^ Mean values with different superscript letters are significantly different (*p* < 0.05) by the Duncan’s multiple-range test. Silymarin, mice treated with 200 mg/kg silymarin; LSA-HN, mice treated with 400 mg/kg *Lotus plumule* alkaloids (Hunan, China); LSA-JX, mice treated with 400 mg/kg *Lotus plumule* alkaloids (Jiangxi, China); LSA-HB, mice treated with 400 mg/kg *Lotus plumule* alkaloids (Hubei, China).

**Table 4:** The levels of AST, ALT and TBIL in the serum from the mice (*N* = 10).

Group	AST (U/L)	ALT (U/L)	TBIL (μmol/L)
Normal	19.32 ± 3.92 ^c^	29.08 ± 7.20 ^d^	14.93 ± 3.93 ^c^
Control	75.50 ± 4.32 ^a^	218.55 ± 21.28 ^a^	40.31 ± 0.82 ^a^
Silymarin	41.92 ± 1.21 ^b^	141.41 ± 13.92 ^c^	16.27 ± 2.41 ^c^
LSA-HN	46.06 ± 1.61 ^b^	174.241 ± 3.43 ^b^	33.97 ± 9.27 ^b^
LSA-JX	53.56 ± 7.70 ^b^	169.43 ± 12.59 ^b^	21.61 ± 2.18 ^b^
LSA-HB	43.75 ± 5.32 ^b^	171.64 ± 13.52 ^b^	31.85 ± 9.85 ^b^

Values presented are the mean ± standard deviation (*N* = 10/group). ^a–d^ Mean values with different superscript letters are significantly different (*p* < 0.05) by the Duncan’s multiple-range test. Silymarin, mice treated with 200 mg/kg silymarin; LSA-HN, mice treated with 400 mg/kg *Lotus plumule* alkaloids (Hunan, China); LSA-JX, mice treated with 400 mg/kg *Lotus plumule* alkaloids (Jiangxi, China); LSA-HB, mice treated with 400 mg/kg *Lotus plumule* alkaloids (Hubei, China).

**Table 5:** The levels of superoxide dismutase (SOD) and malondialdehyde (MDA) in hepatic tissue of mice (*N* = 10).

Group	SOD (U/mg)	MDA (nmol/mg)
Normal	138.65 ± 6.58 ^a^	1.23 ± 0.18 ^d^
Control	52.71 ± 3.07 ^d^	2.69 ± 0.24 ^a^
Silymarin	108.87 ± 5.38 ^b^	1.66 ± 0.19 ^c^
LSA-HN	82.51 ± 4.33 ^c^	2.08 ± 0.16 ^b^
LSA-JX	83.06 ± 5.51 ^c^	1.99 ± 0.17 ^b^
LSA-HB	81.88 ± 3.10 ^c^	2.04 ± 0.12 ^b^

Values presented are the mean ± standard deviation (N=10/group). ^a–d^ Mean values with different superscript letters are significantly different (*p* < 0.05) by the Duncan’s multiple-range test. Silymarin, mice treated with 200 mg/kg silymarin; LSA-HN, mice treated with 400 mg/kg *Lotus plumule* alkaloids (Hunan, China); LSA-JX, mice treated with 400 mg/kg *Lotus plumule* alkaloids (Jiangxi, China); LSA-HB, mice treated with 400 mg/kg *Lotus plumule* alkaloids (Hubei, China).
